# Lycopene sensitizes the cervical cancer cells to cisplatin via targeting nuclear factor-kappa B (NF-κB) pathway

**DOI:** 10.3906/sag-2005-413

**Published:** 2021-02-26

**Authors:** Oktay Halit AKTEPE, Taha Koray ŞAHİN, Gürkan GÜNER, Zafer ARIK, Şuayib YALÇIN

**Affiliations:** 1 Department of Medical Oncology, Faculty of Medicine, Hacettepe University, Ankara Turkey; 2 Department of Internal Medicine, Faculty of Medicine, Hacettepe University, Ankara Turkey

**Keywords:** Lycopene, cervix cancer, cancer treatment, chemotherapy

## Abstract

**Background/aim:**

Lycopene is associated with anticancer effects in various tumor types. However, the exact underlying mechanisms of action of lycopene in human cervical cancer remain to be determined. This study aimed to determine anticancer efficacy and mechanism of lycopene in human cervical carcinoma (HeLa) cells.

**Materials and methods:**

HeLa cells were treated with cisplatin (1 µM) alone, lycopene (10 µM) alone, and in combination for 72 h. The cell viability of HeLa cells was assessed via MTS assay. Western blot was used to analyze the expression levels of the nuclear factor-kappa B (NF-κB), B-cell-associated X protein (Bax), nuclear factor erythroid 2-related factor (Nrf2), and B-cell lymphoma 2 (Bcl-2).

**Results:**

We found that lycopene acts as a synergistic agent with cisplatin in preventing the growth of HeLa cells. The rates of HeLa cells’ viability were 65.6% and 71.1% with lycopene and cisplatin treatment alone compared to the control group, respectively (P < 0.001). The inhibitory effect of cisplatin was enhanced with lycopene addition by declining the cell viability to 37.4% (P < 0.0001). Lycopene treatment significantly increased Bax expression (P < 0.0001) and decreased Bcl-2 expression (P < 0.0001) in HeLa cells. Furthermore, lycopene markedly activated the Nrf2 expression (P < 0.001) and suppressed the NF-κB signaling pathway (P < 0.0001).

**Conclusion:**

Lycopene increases the sensitization of cervical cancer cells to cisplatin via inhibition of cell viability, up-regulation of Bax expression, and down-regulation of Bcl-2 expression. Furthermore, the anticancer effect of lycopene might be also associated with suppression of NF-κB-mediated inflammatory responses, and modulation of Nrf2-mediated oxidative stress. The results of the present study suggest that lycopene and concurrent cisplatin chemotherapy might have a role in improving the treatment of cervical cancer.

## 1. Introduction

Cervical cancer is ranked as the third most frequent gynecological cancer in the United States and the mortality rate is high [1]. Fortunately, the incidence of cervical cancer has decreased as a result of cytological screening (e.g., the Pap smear test), DNA testing for carcinogenic human papillomavirus (HPV) types, and HPV vaccination [2]. However, cervical cancer remains a major problem, with an estimated 569,000 new cancer cases worldwide and 311,000 deaths in 2018 [3]. Current treatment options of cervical cancer include chemotherapy, surgery, and radiotherapy that can be used either alone or in combination. Cisplatin-based combination therapy is the most commonly used chemotherapeutic regimen for advanced carcinomas of the cervix [4]. Nevertheless, common side effects and chemotherapy resistance development significantly impair the effectiveness of cisplatin in the treatment of advanced carcinomas of the cervix [5]. 

The development of drug resistance remains a major barrier to cancer treatment. Although the molecular basis for cancer therapy resistance is not well known, various molecular pathways are considered to play a role. Previous studies have shown that the nuclear factor kappa B (NF-κB) transcription factor is a vital mechanism of chemotherapy resistance [6]. Activation of NF-κB leads to the enhancement of cell proliferation and up-regulation of antiapoptotic genes, both of which contribute to carcinogenesis [7]. Elevated NF-κB activity is a common phenomenon in many types of cancer, notably in cervical cancer [8]. NF-κB also plays a significant role in the progress of platinum-based chemotherapy resistance, such as cisplatin. As a result, NF-κB was considered to be a possible target for current therapies, particularly as an adjuvant to overcome platinum-containing chemotherapy resistance [9]. Another transcription factor, nuclear factor erythroid 2-related factor 2 (Nrf2), plays a critical role in the antioxidative process [10]. Lee et al. suggested that Nrf2 enhances the expression of various antioxidants and detoxification enzymes that play a central role in the mitigation of oxidative stress [11]. Ben-Neriah et al. showed that a wide range of diseases such as autoimmune disorders, neurodegeneration, and cancer was associated with a disparity between Nrf2 and NF-κB pathways [12]. The simultaneous activation and inactivation of Nrf2 and NF-κB are considered novel targets of chemopreventive agents [10]. 

Nutritional factors are widely accepted to be critical in carcinogenesis. Carotenoids are pigments found naturally in the most red-orange-colored fruits and vegetables [13]. Lycopene has been a widely used carotenoid with an unsaturated hydrocarbon chain that can function as an antioxidant [14]. Besides its antioxidant properties, lycopene has been reported to exhibit anticarcinogenic, antiinflammatory, and cardioprotective activities [15]. The anticancer potential of lycopene against certain types of cancer has been shown, including prostate, pancreatic, and breast cancer [16–18]. Although the main anticancer mechanism of lycopene has not been elucidated, it is shown that lycopene induces apoptosis via down-regulation of antiapoptotic protein Bcl-2, up-regulation of the proapoptotic proteins Bim, Bax, and Bid proteins and caspase activation [19]. Furthermore, lycopene has been shown to suppress the NF-κB transcription factor, which plays a pivotal role in the regulation of apoptosis in pancreatic, prostate, and breast cancer cell lines [18,20]. However, there is no previous study on the influences of lycopene on human cervical carcinoma (HeLa) cells and the corresponding mechanism. Therefore, this study aimed to assess the synergistic mechanism of cisplatin and lycopene and the anticancer properties of lycopene on cervical cancer HeLa cells through evaluating its effect on proliferation, apoptosis, and inflammatory signaling pathways, namely NF-κB and Nrf2. 

## 2. Materials and methods

### 2.1. Cell culture and reagents

A HeLa cervical cancer cell line was obtained from the American Type Culture Collection (Manassas, VA, USA). Cisplatin was supplied by Sigma (St. Louis, MO, USA) and lycopene was provided by DSM Nutrition (İstanbul, Turkey). HeLa cells were grown and maintained in an appropriate RPMI-1640 medium supplemented with 10% fetal bovine serum, 100 µg/mL streptomycin, 2 mM L-glutamine, and 100 U/mL penicillin G. Cell incubation was performed at 37 °C in a humidified incubator with an atmosphere containing 5% CO2. No growth factor has been added to the media of the cell culture. In phosphate-buffered saline (PBS), cisplatin was dissolved to form a 1 mM stock solution and final working concentrations.

### 2.2. Treatment protocol and cell viability assay

A 3- (4,5-dimethylthiazol-2-yl) -5- (3-carboxymethoxyphenyl) -2- (4-sulfophenyl) -2H-tetrazolium (MTS) colorimetric assay was used to determine the viability of cells. In brief, cells were seeded in 96-well plates at a concentration of 104 cells per well and incubated overnight to allow cells to attach. After incubation, the cells were treated with lycopene alone (10 µM), cisplatin alone (1 µM), and in combination for 72 h. The cells were incubated at 37 °C in 5% CO2 for 2 h after 72 h of total treatment. For assay a 1 mg/mL MTS reagent (Sigma, St. Louis, MO, USA) was added to each well. The absorbance of the solution was estimated using a microplate reader (BioTek Instruments, Winooski, VT, USA) at 490 nm. The sample readings were calculated by subtracting the mean of background absorbances. Viability was calculated concerning control cells (%). MTS assay was performed at least four times.

### 2.3. Western blot analysis

The HeLa cell extracts were obtained with PBS-1% TritonX-100 (Sigma) containing a lysis buffer, and 20 µg of protein preparation was loaded onto 10% acrylamide gels in each lane. Proteins in samples were fractionated by electrophoresis and migrated on 10% SDS-PAGE, then transferred to a nitrocellulose membrane. Blocking was carried out using 5% dry milk for 2 h. Then, membranes were incubated overnight with diluted (1:1000) primary antibody, anti-Nrf2, anti-Bcl-2, anti-Bax, and anti-NF-κB p65 (Santa Cruz, CA, USA) at 40 °C. Antibody labeling was detected by incubation with a secondary antibody (HRP-linked goat antimouse IgG, Abcam, Cambridge, UK). A monoclonal mouse β-actin antibody (A5316, Sigma) was used to control the loading of protein. Band quantification analysis was done using ImageJ software (NIH, Bethesda, USA). Standardization of results was achieved using the β-actin expression as a percentage of control in each group. Blots were performed in triplicate to confirm the reproducibility of data.

### 2.4. Statistical analysis

All measures were presented as group mean ± SD, which were replicated four times. Experimental data were carried out with one-way ANOVA using the General Linear Model (SPSS version 23.0, Chicago, IL, USA). Tukey’s post hoc test was applied to clarify group mean changes. Statistical significance defined as P < 0.05 was indicated by a number sign (#). In addition, P < 0.01 and P < 0.001 were marked with 2 (##) and 3 (###) number signs, respectively, compared to the control group in the figures. GraphPad Prism 7.0 (GraphPad Software) was used for data graphing.

## 3. Results

### 3.1. Lycopene enhances the growth inhibitory effect of cisplatin on the proliferation of HeLa cells

**Figure 1:**
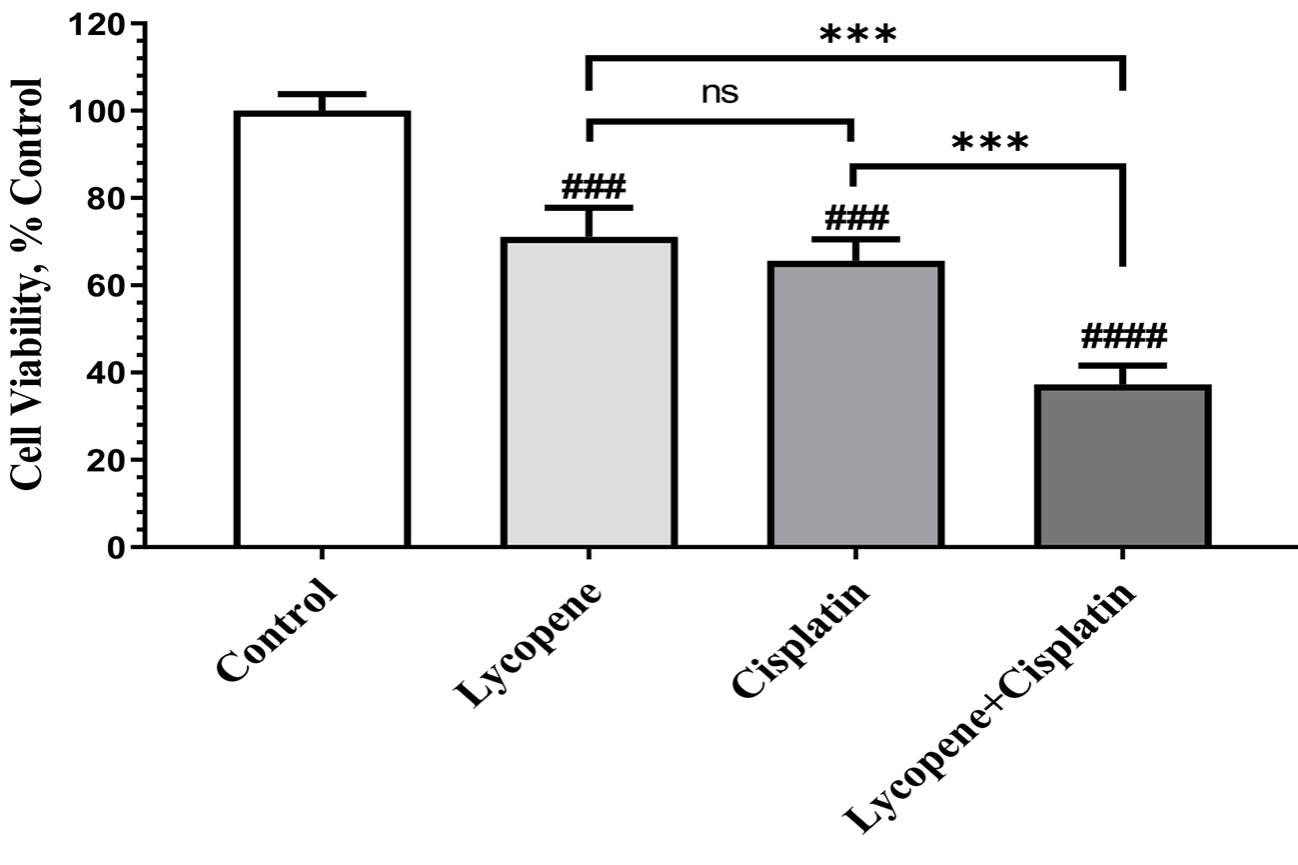
The effects of lycopene treatment on the proliferation of HeLa cell lines. The data are described as having cell viability relative to vehicle control of treated cell and shown as mean ± SD of four independent experiments (ANOVA and Tukey’s post hoc test statistical significance between groups is shown by # P < 0.05, ## P < 0.01, ### P < 0.001, #### P < 0.0001 compared as control group; *** P < 0.001, **** P < 0.0001 shows comparison between each treatment group, and ns means not significant.).

The effects of treatment of lycopene (10 µM), cisplatin (1 µM), and the combination of both for 72 h on the viability of HeLa cervical carcinoma cells were measured through MTS assay. MTS analysis presented that lycopene and cisplatin treatment alone decreased the viability of HeLa cells to 65.6% and 71.1%, respectively (P < 0.001, Figure 1). When cells were treated in combination with lycopene and cisplatin, lycopene improved the inhibitory effect of cisplatin by declining the cell viability to 37.4% compared to the control group (P < 0.0001, Figure 1). The combination treatment with cisplatin and lycopene was found to be more efficient than cisplatin and lycopene alone treatment to inhibit cell growth at 72 h in HeLa cells. Above all, these results indicate that lycopene increases the sensitivity of HeLa cells to cisplatin.

### 3.2. Lycopene sensitizes HeLa cells to cisplatin-induced apoptosis markers 

**Figure 2:**
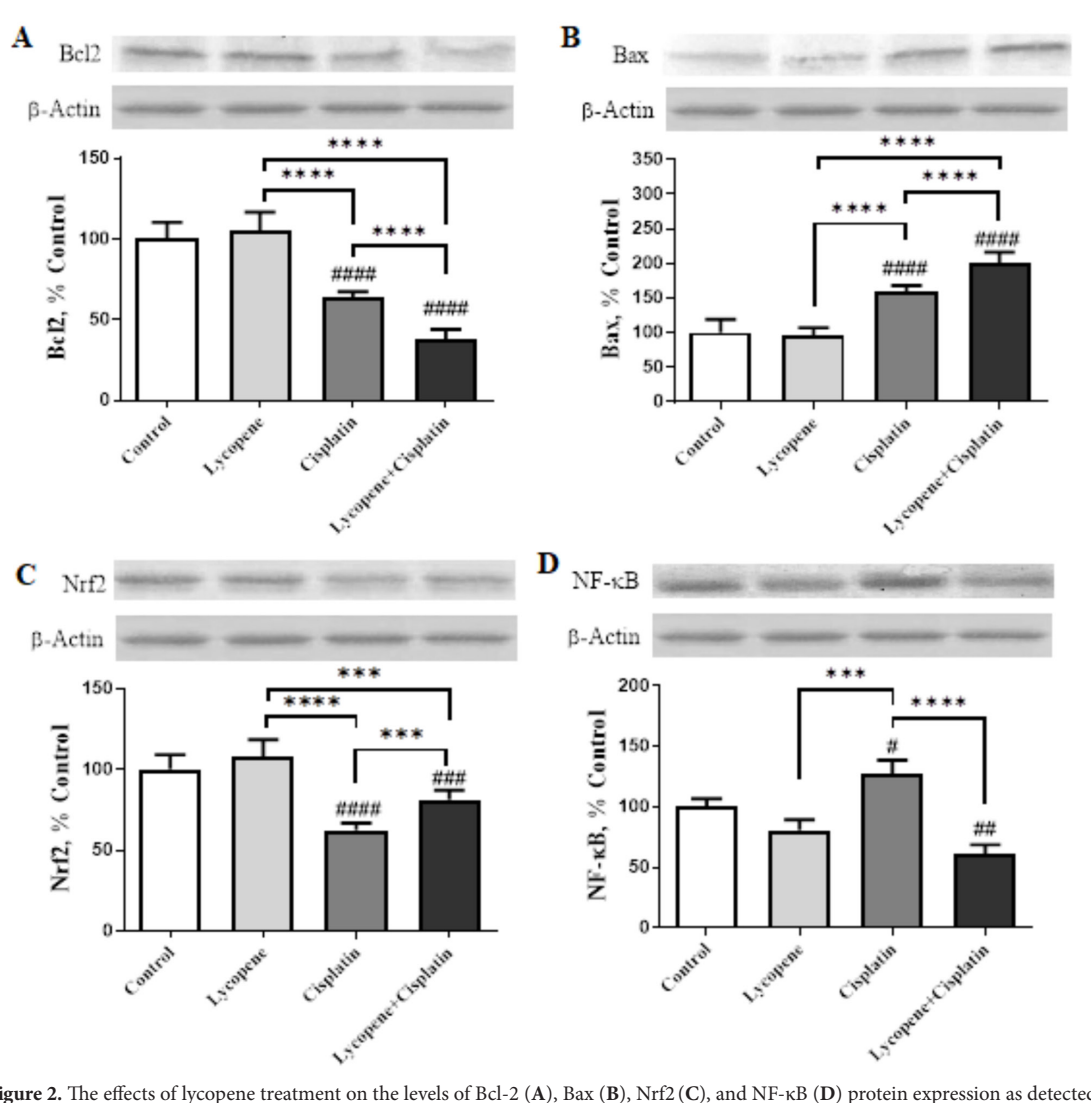
The effects of lycopene treatment on the levels of Bcl-2 (A), Bax (B), Nrf2 (C), and NF-κB (D) protein expression as detected by Western blotting analysis of HeLa cells. The band intensities were measured using densitometric analysis. The data were presented as a ratio of treatment value to control value set at 100%. The bar represents the mean and standard error of the mean. Western blots were repeated at least four times. β-actin was used as a loading control protein for Western blotting analysis (ANOVA and Tukey’s post hoc test statistical significance between groups is shown by # P < 0.05, ## P < 0.01, ### P < 0.001, #### P < 0.0001 relative to control group; *** P < 0.001, **** P < 0.0001 shows comparison between each treatment group).

To evaluate the possible mechanism responsible for the anticancer effects of lycopene, we investigated whether cisplatin in combination with lycopene induced more apoptosis than either agent alone. HeLa cells treated with lycopene (10 µM), cisplatin (1 µM), and combination of both for 72 h and apoptosis-related proteins Bcl-2 and Bax levels were analyzed by western blotting. Lycopene and concurrent cisplatin chemotherapy enhanced the cisplatin-induced properties by reducing the antiapoptotic Bcl-2 protein (P < 0.0001, Figure 2A) and increasing proapoptotic Bax levels (P < 0.0001, Figure 2B) compared to cisplatin-only treated cells. 

### 3.3. Lycopene suppresses NF-κB activation and induces Nrf2 activation in HeLa cells 

The level of Nrf2 was significantly reduced in the cisplatin-only treated HeLa cells compared with those expressed in the control HeLa cells. It was found that lycopene increased Nrf2 expression in lycopene-only treated cells compared to control cells. The combination group had a significantly higher Nrf2 level than the cisplatin-only treated cells group (P < 0.001, Figure 2C). Lycopene decreased NF-κB activation, which is increased by cisplatin in HeLa cells. As shown in Figure 2D, cisplatin-only treated cells had increased the NF-κB p65 level, while lycopene-only treated cells had reduced this protein compared to control. The NF-κB p65 expression level was further reduced by adding lycopene to cisplatin only treated cell group (P < 0.0001). 

## 4. Discussion 

Chemoprevention is considered one of the most mentioned factors in combating cancer. Lycopene, other than being a color pigment naturally available in tomatoes, is proven to drastically decrease the risk of cancer thanks to epidemiologic and cell culture studies. Even though the anticarcinogenic activity and structure of lycopene have been studied therapeutically on many types of tumors, its impact on cervical cancer, a condition with a high mortality rate on women, has yet to be fully understood. In the current study, we determined whether lycopene alone will show an effect on HeLa cells, and by which mechanism and how it will affect the action of cisplatin, one of the most frequently used chemotherapeutic drugs in cervical cancer. The mechanism of the inhibitory effect of lycopene on HeLa cells was investigated at the molecular level by determining Bcl-2, Bax, NF-κB, and Nrf2 protein expression levels. To our knowledge, this is the first research to evaluate the role of lycopene in Nrf2 and NF-κB pathways in a human cervical cancer cell line (HeLa).

 Apoptosis can be defined as biochemical and morphological cellular changes that occur via caspase-mediated pathways in response to various fatal stimuli. On the other hand, cancerous cells can become resistant to apoptosis through a wide range of mechanisms. There are two main apoptotic pathways, named extrinsic and intrinsic, which are responsible for carcinogenesis regulation [21]. The intrinsic pathway is activated through the decomposition of the mitochondrial membrane and the release of cytochrome c that functions in the activation of caspase-9 [22]. Mitochondrial membrane stabilization is ensured through proapoptotic (Bcl-XL and Bcl-2) and antiapoptotic Bcl-2 (Bax, Bim, and Bid) family members with different functions [23]. Many reports have presented that lycopene induces apoptosis by activating the intrinsic pathway [24,25]. In a study conducted by Sahin et al., it was shown that the risk of chemically-induced breast cancer was decreased in rats in vivo through treatment with a lycopene and genistein combination that ensured a reduction of Bcl-2 and an increase in Bax proteins [24]. Application of lycopene to hormone-refractory prostate cancer cell lines in a dose-dependent manner has resulted in a decrease of Bcl-2 expression, an increase in Bax proteins, and induction of apoptosis by causing cycle distribution changes [25]. Similar to these findings, our results showed that lycopene treatment, in addition to cisplatin, led to a decreased Bcl-2 level and an increased Bax level, suggesting that lycopene enhances the potency of cisplatin by affecting apoptosis-related protein levels on HeLa cells. It is quite likely in the future to monitor drastic changes in many tumors by inducing apoptosis through the intrinsic pathway with lycopene and anticancer drugs. 

NF-κB undertakes the role of a bridge between inflammation and cancer together with the regulation of genes that have functions in immunity, inflammation, and tumor development as a transcription factor [12]. Under normal circumstances, NF-κB is available in an inactive form and attached to IκBα within the cellular cytoplasm. Phosphorylation of IκBα by varying stimuli leads to the activation of NF-κB. However, NF-κB remains constantly active in a cancerous cell by inducing the expression of the apoptosis-inhibiting genes such as cIAP1, cIAP2, and survivin, and leads to uncontrolled cellular growth [26,27]. Lycopene treatment decreases RAS-dependent activation of NF-κB, regulating the effects of the cell cycle and apoptosis-related proteins such as cyclin D1, p21, p27, Bax, and Bcl-2 [28]. It was found that combination treatment of ursolic acid, an NF-κB inhibitor, with taxane and cisplatin in HeLa cells decreased the dose of chemotherapy drugs required to produce the same effect and resulted in a synergistic increase in the formation of chemotherapy agent-induced apoptosis [29]. Lycopene has been monitored to decrease the expression of prosurvival genes by causing NF-κB inhibition in pancreatic cancerous cells [18]. Additionally, lycopene decreases the invasiveness of human hepatoma cells by reducing the attachment capacity of NF-κB and SP-1 (specificity protein) to DNA, thus allowing the down-regulation of the matrix metalloproteinase (MMP)-9 enzyme [30]. These studies show that NF-κB has a key role in cellular survival, and NF-κB inhibition is an essential target in combating cancer. In this study, we found that the highest NF-κB activation was obtained in the cisplatin group, while the lowest NF-κB activation was achieved with the combined use of lycopene and cisplatin. In other words, additional inhibition of NF-κB activation by lycopene has made the combination of lycopene and cisplatin treatment even more effective. 

As a transcription factor, Nrf2 was first described by its capacity to bind to NF-E2/AP-1 repeat region in the promoter region of the ß-globin gene [31] and has a vital role in the induction of phase-II enzymes with key detoxification and antioxidant features against reactive oxygen radicals and potential carcinogens [10,32]. The transcriptional up-regulation of these phase-II enzymes are ensured via cis-acting DNA series that are titled as antioxidant response element (ARE) located in the promoter regions of these enzymes. Nrf2 is found in a complex form with an inhibitory Keap1 protein in cellular cytoplasm under normal conditions. After exposure to oxidative stress, Nrf2 is separated from the complex and passes to the nucleus to regulate the target genes by binding to antioxidant response elements along with other transcription factors [33]. Interestingly, Nrf2 has been observed to possess quite different features from each other depending on the stage of the carcinogenesis process. For example, it functions as a tumor-suppressive factor by binding to ARE and thus regulating cellular detoxification and antioxidant response in the early stage of pancreatic carcinogenesis [34]. The tumor may become even more aggressive as a result of the aberrant Nrf2 overexpression caused by Keap 1 mutation and silencing, which are frequently observable in the later stage of the above-mentioned carcinogenesis [35]. Carotenoids realize their antioxidant features by increasing cytoprotective enzyme levels that play an active role in cellular oxidative stress response by enabling Nrf2 activation, a primary ARE transcription factor [36]. In a study in which lycopene was used for preventive strategies, it was observed that lycopene reduced 12-dimethylbenz[a]anthracene-induced hamster buccal pouch carcinogenesis by activating detoxification pathways and ultimately inhibiting oxidative stress [37]. In this study, we showed that the combination treatment of lycopene and cisplatin increased Nrf2 more than cisplatin treatment alone. Even though it is not quite clear how lycopene induces the nuclear translocation of Nrf2, a previous study showed that the interaction of lycopene with cysteine residues of Keap1 resulted in Nrf2 activation by its release from the Keap1-Nrf2 complex [38]. Likewise, the enhancement of the translocation of Nrf2 to the nucleus by lycopene-derived metabolites via affecting the upstream signal pathways is another possible mechanism [19]. Due to key detoxifying and antioxidant functions in the carcinogenesis process, evaluation of Nrf2 as a molecule that is attractive and possibly efficient for new cancer treatment strategies appears to be an inevitable approach and reality. Considering the dual function of Nrf2 in the carcinogenesis process, both Nrf2 induction and Nrf2 inhibition might be targets of therapeutic medication administration.

In this study, we revealed that lycopene acts synergistically with cisplatin to prevent the growth of cervical cancer cell lines. The findings of the present study showed that cisplatin treatment is potentiated with lycopene in HeLa cells by modulation of Bcl-2, Bax, NF-κB, and Nrf2, which are important for cell survival and apoptosis. Together, the results suggest that lycopene and concurrent cisplatin chemotherapy could be used to improve the treatment of cervical cancer by reducing cell survival and increasing apoptosis. Therefore, this study can give a key clue to the use of lycopene together with chemotherapeutic agents. Future in vitro and animal studies are necessary to examine the concurrent use of cisplatin and lycopene in cervical cancer given the high rates of new cervical cancer and related side effects of conventional treatment modalities.

## 5. Conclusion

In conclusion, lycopene further strengthened the positive effect of cisplatin on human cervical cancer cells by regulating apoptosis proteins and transcription factors. However, many preclinical and clinical studies are needed to examine the effects of lycopene in the prevention and treatment of cervical cancer. 

## Funding

The authors did not receive financial support for this research.
